# The effects of the COVID-19 pandemic on dengue cases in Malaysia

**DOI:** 10.3389/fpubh.2023.1213514

**Published:** 2023-08-24

**Authors:** Nuur Hafizah Md Iderus, Sarbhan Singh Lakha Singh, Sumarni Mohd Ghazali, Asrul Anuar Zulkifli, Nur Ain Mohd Ghazali, Mei Cheng Lim, Lonny Chen Rong Qi Ahmad, Mohamad Nadzmi Md Nadzri, Cia Vei Tan, Ahmed Syahmi Syafiq Md Zamri, Chee Herng Lai, Nur Shuhada Nordin, Mohd Kamarulariffin Kamarudin, Ming Keong Wan, Norhayati Mokhtar, Jenarun Jelip, Balvinder Singh Gill, Nur Ar Rabiah Ahmad

**Affiliations:** ^1^Biomedical Epidemiology Unit, Special Resource Centre, Institute for Medical Research, National Institutes of Health, Ministry of Health, Shah Alam, Malaysia; ^2^Vector-Borne Disease Sector, Disease Control Division, Ministry of Health, Putrajaya, Malaysia; ^3^Special Resource Centre, Institute for Medical Research, National Institutes of Health, Ministry of Health, Shah Alam, Malaysia

**Keywords:** COVID-19, dengue, correlation, incidence, demographic, spatial distribution

## Abstract

**Background:**

Globally, the COVID-19 pandemic has affected the transmission dynamics and distribution of dengue. Therefore, this study aims to describe the impact of the COVID-19 pandemic on the geographic and demographic distribution of dengue incidence in Malaysia.

**Methods:**

This study analyzed dengue cases from January 2014 to December 2021 and COVID-19 confirmed cases from January 2020 to December 2021 which was divided into the pre (2014 to 2019) and during COVID-19 pandemic (2020 to 2021) phases. The average annual dengue case incidence for geographical and demographic subgroups were calculated and compared between the pre and during the COVID-19 pandemic phases. In addition, Spearman rank correlation was performed to determine the correlation between weekly dengue and COVID-19 cases during the COVID-19 pandemic phase.

**Results:**

Dengue trends in Malaysia showed a 4-year cyclical trend with dengue case incidence peaking in 2015 and 2019 and subsequently decreasing in the following years. Reductions of 44.0% in average dengue cases during the COVID-19 pandemic compared to the pre-pandemic phase was observed at the national level. Higher dengue cases were reported among males, individuals aged 20–34 years, and Malaysians across both phases. Weekly dengue cases were significantly correlated (*ρ* = −0.901) with COVID-19 cases during the COVID-19 pandemic.

**Conclusion:**

There was a reduction in dengue incidence during the COVID-19 pandemic compared to the pre-pandemic phase. Significant reductions were observed across all demographic groups except for the older population (>75 years) across the two phases.

## 1. Introduction

A novel coronavirus (COVID-19) was first discovered late in December 2019 which subsequently resulted in the COVID-19 pandemic which was declared by World Health Organization (WHO) on 11 March 2020 (1). As the COVID-19 pandemic progressed, it affected the transmission dynamics of several other infectious diseases globally (2, 3). Dengue is one of the infectious diseases that was affected during the COVID-19 pandemic, wherein a reduction in dengue case trends during the pandemic was reported (4, 5).

Dengue is endemic in over 100 countries in the tropical and subtropical regions including Africa, the Americas, the Eastern Mediterranean, South-East Asia and the Western Pacific. Over the last two decades, the number of dengue cases reported worldwide by the WHO increased drastically from 505,430 cases in 2000 to over 5.2 million in 2019 (6). Similarly, in Malaysia, dengue cases have increased by 1,561% from the year 1995 to 2014, which makes dengue one of the highest burdens in the country (7).

A study conducted in 2017 projected that there would be an increasing trend of the dengue incidence rate of 628 to 940 per 100,000 population from 2020 to 2040 in Malaysia (8). However, in the year 2020, when the COVID-19 pandemic occurred, the incidence of dengue was lower compared to what was projected with an incidence of 277 per 100,000 population. Several studies reported that the COVID-19 movement control measures had resulted in the reduction of dengue cases in the year 2020 (9, 10). However, there are no published studies to describe the effects of the COVID-19 pandemic on dengue cases at the national, state and district levels in Malaysia. In addition, to date, there are no studies that have examined and compared the distribution of dengue cases by demographic subgroups for the pre and during COVID-19 pandemic phases in Malaysia. This would be important to determine and quantify the effects of the pandemic on dengue.

Therefore, this study aims to describe the effect of the COVID-19 pandemic on the geographical and demographic distribution of dengue cases and incidence in Malaysia from 2014 to 2021. Furthermore, this study also examines the correlation between COVID-19 and dengue cases at the national and state levels during the COVID-19 pandemic. Findings from the study would be important in assisting the control and management of dengue in Malaysia during the endemic phase of COVID-19.

## 2. Methods

### 2.1. Data source

#### 2.1.1. Dengue data

Since the implementation of the Prevention and Control of Infectious Disease Act 1988 (Act 342) in Malaysia, it has been mandatory to report all suspected dengue cases within 24 h to the nearest district health office. All suspected dengue cases which meet both the clinical case definition and laboratory confirmation of dengue fever are registered as confirmed dengue cases in the eDengue database from 2014 onwards (11). Dengue case data from January 2014 to December 2021 were sourced from eDengue and aggregated by epidemiological weeks at national, state and district levels. In addition, demographic variables such as gender, age, ethnicity and nationality from January 2014 to December 2021 were also sourced from eDengue. All case identifiers were removed and data was anonymised.

#### 2.1.2. COVID data

COVID-19 case data were sourced from the official MOH open-source GitHub repository from January 2020 to December 2021 (12). A total of 2,761,472 anonymized confirmed COVID-19 cases that met the clinical case definition and laboratory confirmation of COVID-19 were obtained at the national and state levels in Malaysia and aggregated by epidemiological week (13, 14).

#### 2.1.3. Population data and shape files

Population data were obtained from the Department of Statistics Malaysia which consists of mid-year population data for each age group, gender, nationality, and ethnicity for the years 2014 to 2021. Malaysia shape files consisting of state and district boundaries were sourced from the Malaysia Geospatial Data Infrastructure (MyGDI).

### 2.2. Data analysis

Population data, dengue and COVID-19 cases were manually extracted and entered into Microsoft Excel for data pre-processing (check for missing values) and storage. Data were analyzed using the Statistical Package for the Social Sciences (SPSS) version 26.0 release 2019 by International Business Machines, IBM Corp (15). The annual dengue case incidence from 2014 to 2021 at the national, state and district levels were determined by dividing the annual dengue cases over the mid-year population of the respective year and was presented graphically and in a tabular format.

Subsequently, the analysis was conducted in two phases, which is the pre-COVID-19 pandemic from 2014 to 2019, and during COVID-19 pandemic from 2020 to 2021, which will be referred to as the ‘pre-pandemic’ and ‘pandemic’ phases henceforth, respectively. The average annual national, state and district dengue case incidence was calculated and compared for the pre (2014–2019) and pandemic (2020–2021) phases. Dengue case incidence during the pre and pandemic phases were presented graphically and as a choropleth map. In addition, the average dengue case incidence by demographic subgroups (age groups, gender, nationality and ethnics) for the state and national level were calculated and compared for the pre (2014–2019) and pandemic (2020–2021) phases, respectively, and was presented graphically and in a tabular format. The incidence rate ratio (IRR) was estimated by dividing the average incidence rate during the pandemic by the average pre-pandemic rate along with its 95% confidence interval (CI). An IRR is interpreted as significant if its 95% CI does not contain the value 1 (16).

In addition, a correlation analysis between weekly dengue and COVID-19 cases during the pandemic phase (2020 to 2021) was performed to determine the association between the two diseases at the state and national levels. The dataset was tested for normality prior to performing correlation analysis by using the Shapiro–Wilk test. As the data were not normally distributed (value of *p* = <0.001), Spearman Rank correlation (*ρ*) was performed to identify the monotonic relationship and correlation direction between the weekly dengue and COVID-19 cases during the COVID-19 pandemic. The magnitude of change for two variables is either in the same or in the opposite direction indicated by a positive or negative value of the correlation coefficient with a value of p less than 0.05 indicating significant correlations. The classification of strength of the relationship was according to Schober Patrick’s study no association (*ρ* = 0) to a perfect positive relationship (*ρ* = +1) and a perfect negative relationship (*ρ* = −1) (17).

## 3. Results

### 3.1. Trends of dengue case incidence at the national and state level in Malaysia from 2014 to 2021

**Figure 1:**
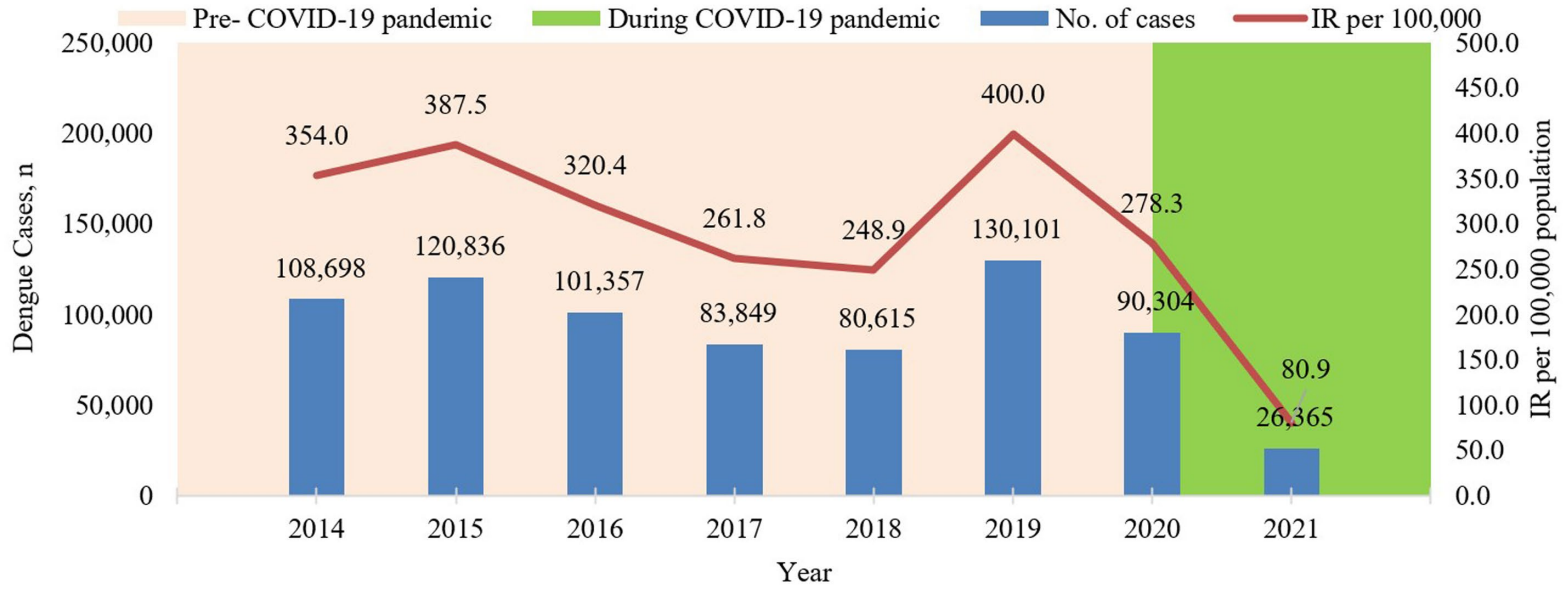
Annual dengue cases and incidence rate per 100,000 population in Malaysia from 2014 to 2021.

A total of 742,125 confirmed dengue cases were reported in Malaysia from the year 2014 to 2021 as shown in Figure 1. The highest incidence of dengue was recorded in the year 2019 at 400.0 per 100,000 population (130,101 cases), while the lowest incidence was recorded in 2021 at 80.9 per 100,000 population (26,365 cases) (Figure 1). Dengue incidence peaked in the year 2015 and 2019 and subsequently decreased in the year 2020 and 2021 Decreasing dengue case incidence was observed during the pandemic phase. As compared to 2019, which had recorded the highest dengue case incidence, there was a reduction in dengue case incidence of 30.4 and 79.8% for the years 2020 and 2021, respectively.

**Figure 2:**
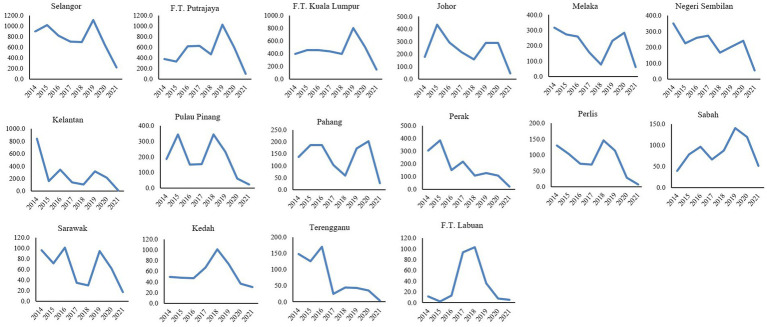
Incidence rate of dengue (per 100,000 population) by state, 2014–2021, Malaysia.

Selangor state had reported the highest dengue incidence rate compared to the other states throughout the year 2014 to 2021 with a median of 765.4 per 100,000 population and the highest incidence of dengue was recorded in the year 2019 with 1115.0 per 100,000 population (72,543 cases). The lowest incidence was recorded in Federal Territory (F.T.) Labuan in 2015 with 2.0 per 100,00 population (2 cases) (Table 1). At the states level, dengue -incidence peaked in the years 2015 and 2019 across the majority of the states except for Kedah, F.T. Labuan and F.T. Putrajaya for the year 2015 and Kedah, Perak, Perlis, Pulau Pinang, Terengganu and F.T. Labuan for the year 2019, respectively, (Figure 2). Decreasing dengue incidence was observed across all states in the year 2020 and 2021 as shown in Table 1.

**Table 1:** Dengue case incidence rate per 100,000 population by state, Malaysia, 2014 to 2021.

State/Year	Incidence rate per 100,000 population								
	2014	2015	2016	2017	2018	2019	2020	2021	Median
Selangor	897.0	1023.0	821.0	709.8	700.4	1115.0	638.1	224.4	765.4
F.T. Putrajaya	383.2	331.3	619.7	630.9	466.0	1029.9	597.1	100.7	531.6
F.T. Kuala Lumpur	395.6	452.5	454.8	434.9	398.8	805.4	503.5	150.3	443.7
Johor	177.6	436.1	291.4	214.6	157.0	289.1	289.8	44.2	251.8
Melaka	317.5	272.2	258.1	156.1	78.3	232.2	284.8	60.8	245.2
Negeri Sembilan	350.9	225.4	259.4	274.2	166.0	204.7	240.9	53.9	233.2
Kelantan	838.7	161.9	344.0	137.5	104.8	318.7	217.0	12.3	189.4
Pulau Pinang	187.2	343.3	150.0	153.7	344.4	232.9	59.9	23.3	170.4
Pahang	136.9	186.6	187.5	102.6	59.5	171.9	202.4	26.7	154.4
Perak	306.0	383.7	152.2	217.0	109.3	128.6	106.8	22.1	140.4
Perlis	129.3	103.8	72.9	69.4	145.6	113.4	28.1	7.3	88.4
Sabah	39.6	78.1	96.5	66.4	87.8	140.3	119.3	51.7	82.9
Sarawak	96.5	71.2	101.3	34.4	29.5	94.4	61.8	17.3	66.5
Kedah	49.2	47.7	46.9	66.7	101.2	73.0	36.6	29.9	48.4
Terengganu	148.0	125.3	169.8	24.2	44.8	43.6	35.1	4.2	44.2
F.T. Labuan	11.7	2.1	13.4	93.2	103.0	36.3	7.4	5.2	12.6

### 3.2. Correlation analysis

**Figure 3:**
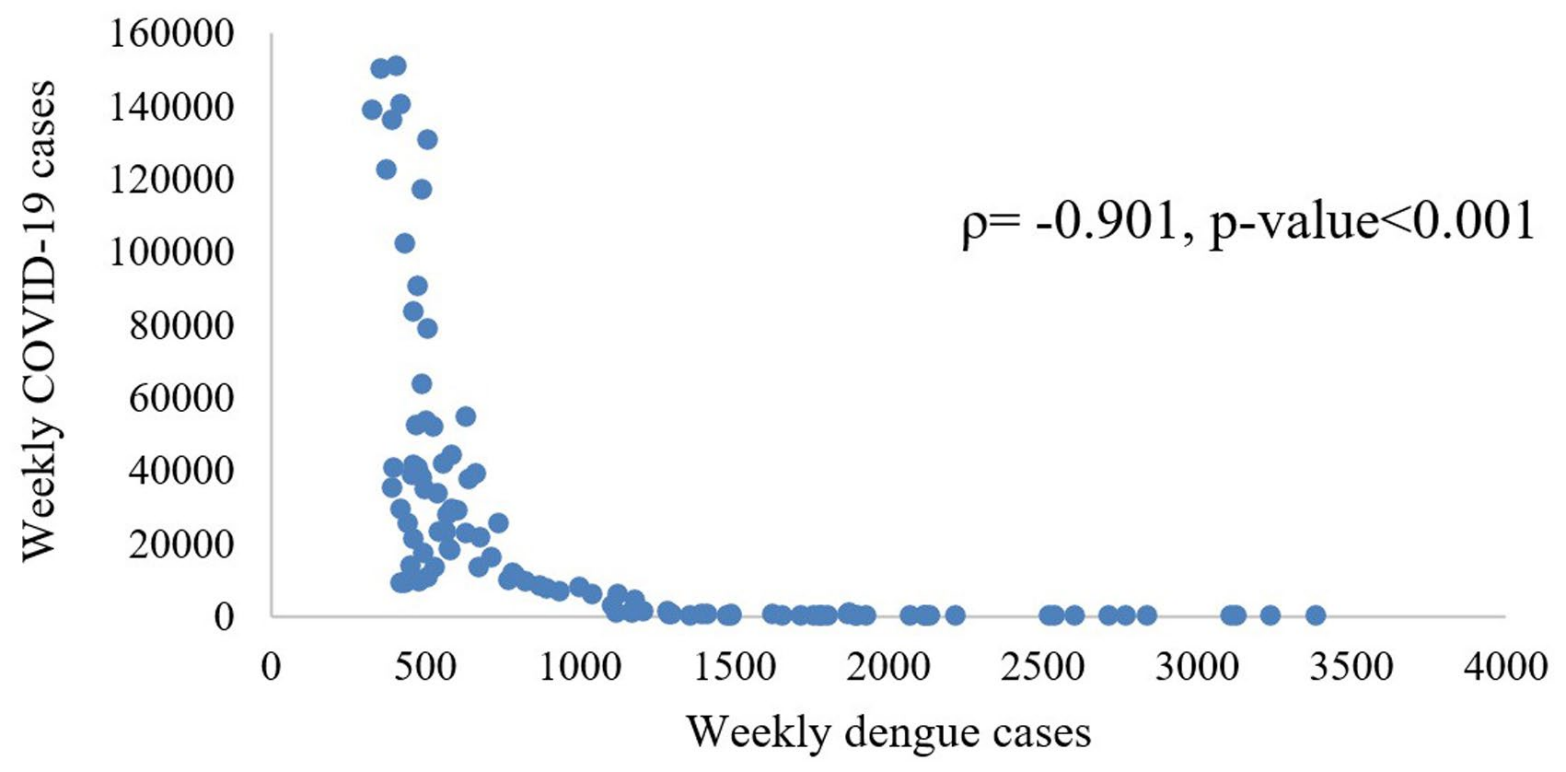
Scatter plot of weekly COVID-19 and dengue cases, Malaysia, 2020–2021.

At the national level, weekly dengue cases were negatively correlated with weekly COVID-19 cases with a significant correlation coefficient of −0.901 (Figure 3; Table 2). Similarly, all states also showed negative correlations between weekly dengue and COVID-19 cases with significant correlation coefficients ranging from −0.378 to −0.897 wherein increasing weekly COVID-19 cases indicates lower weekly dengue cases. The correlation analysis is shown in Table 2.

**Table 2:** Correlation of weekly COVID-19 cases and dengue cases.

State	2020–2021	
	*n* = 105 weeks	
	Correlation, ρ	Value of *p*
Johor	−0.897**	<0.001
Kedah*	−0.623**	<0.001
Kelantan	−0.877**	<0.001
Melaka	−0.861**	<0.001
Negeri Sembilan	−0.800**	<0.001
Pahang	−0.891**	<0.001
Perak	−0.836**	<0.001
Perlis	−0.378**	<0.001
Pulau Pinang	−0.642**	<0.001
Sabah	−0.754**	<0.001
Sarawak	−0.820**	<0.001
Selangor	−0.818**	<0.001
Terengganu	−0.796**	<0.001
F.T. Kuala Lumpur	−0.771**	<0.001
F.T. Putrajaya	−0.720**	<0.001

*Weekly dengue and COVID-19 cases in year 2020 were used in the analysis due to small number of observations. **Significant at value of *p* < 0.05.

### 3.3. Distribution of dengue case incidence for pre and pandemic phases at the national, state and district level in Malaysia

**Figure 4:**
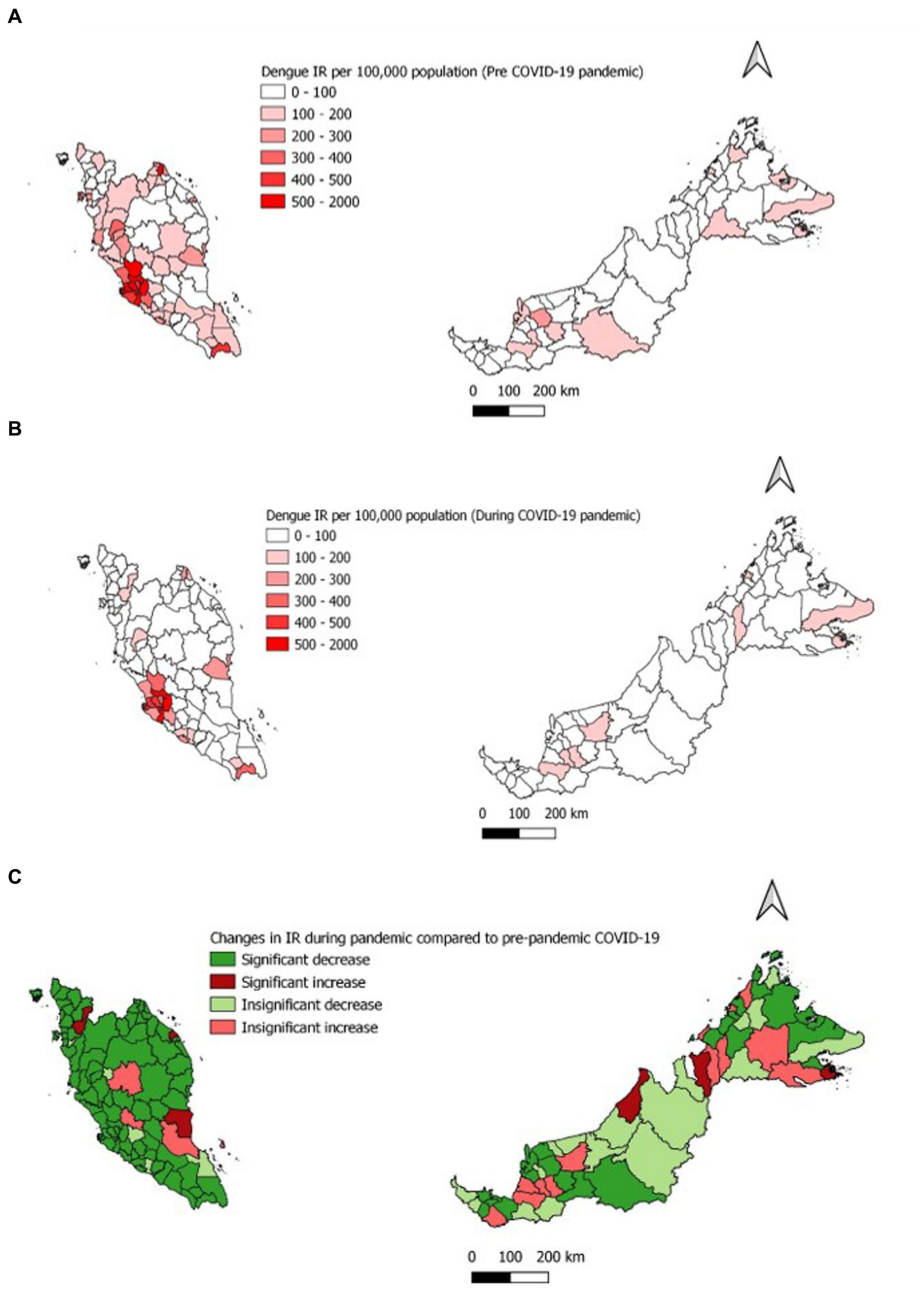
Average dengue incidence rate per 100,000 population **(A)** Pre- COVID-19 pandemic **(B)** During COVID-19 pandemic **(C)** Change in the average IR during COVID-19 pandemic.

Dengue case incidence was reported at 328.8 and 179.6 (per 100, 000 population) for the pre and pandemic phases, respectively, at the national level. This corresponds to a 149.2 reduction in dengue case incidence at the national level during the pandemic compared to the pre-pandemic phase. The incidence rate ratio showed a significantly lower number of dengue infections during the pandemic compared to pre-pandemic phase (IRR = 0.55, 95%CI: 0.54–0.56) as shown in Table 3. Overall, the dengue case incidence was lower in all states (except for Sabah) during the pandemic compared to the pre-pandemic phase with Selangor reporting the highest reduction at 446.4 (Table 3). A similar finding was reported at the district levels wherein 82.1% of districts showed a significantly decreased in dengue case incidence during the pandemic compared to the pre-pandemic phase, with Petaling districts reporting the highest reduction in dengue case incidence at 548.0 (Figure 4).

**Table 3:** Average dengue case incidence pre and during pandemic per 100,000 population and rate ratio by state, Malaysia.

State	Total cases		Standard deviation		Average incidence rate per 100,000 population		Difference of pre and during pandemic incidence rate	Incidence rate ratio (95% confidence interval)
	Pre-pandemic	During pandemic	Pre-pandemic	During pandemic	Pre-pandemic	During pandemic		
Malaysia	625,456	116,669	63.36	139.6	328.8	179.6	−149.2	0.55 (0.54,0.56)*
Johor	57,397	13,397	102.27	173.7	260.9	167.0	−94.0	0.64 (0.62,0.66)*
Kedah	8,215	1,424	21.23	4.7	64.1	33.3	−30.8	0.52 (0.47,0.57)*
Kelantan	33,953	4,112	273.59	144.7	317.6	114.6	−202.9	0.36 (0.34,0.38)*
Melaka	11,817	3,454	87.19	158.3	219.1	172.8	−46.3	0.79 (0.74,0.84)*
Negeri Sembilan	16,318	3,540	64.04	132.2	246.8	147.4	−99.4	0.6 (0.57,0.64)*
Pahang	13,783	3,648	51.65	124.2	140.8	114.5	−26.2	0.81 (0.76,0.86)*
Perak	32,141	3,222	108.94	59.8	216.1	64.5	−151.6	0.3 (0.28,0.32)*
Perlis	1,590	101	30.33	14.7	105.7	17.7	−88.0	0.17 (0.13,0.23)*
Pulau Pinang	24,418	1,449	89.25	25.9	235.2	41.6	−193.7	0.18 (0.17,0.19)*
Sabah	19,487	5,841	33.62	47.8	84.8	85.5	0.7	1.01 (0.96,1.06)
Sarawak	11,690	1,942	32.17	31.5	71.2	39.5	−31.6	0.55 (0.51,0.59)*
Selangor	332,312	60,376	167.71	292.6	877.7	431.3	−446.4	0.49 (0.48,0.5)*
Terengganu	6,536	453	62.4	21.9	92.6	19.7	−72.9	0.21 (0.18,0.24)*
F.T. Kuala Lumpur	52,363	12,930	156.44	249.8	490.3	326.9	−163.4	0.67 (0.65,0.69)*
F.T. Labuan	255	12	44.04	1.5	43.3	6.3	−37.0	0.15 (0.06,0.35)*
F.T. Putrajaya	3,181	768	253.01	351	576.8	348.9	−227.9	0.6 (0.53,0.68)*

*Significant incidence rate ratio.

### 3.4. Demographic distribution of dengue case incidence and percentage for pre and pandemic phases at the national and state level in Malaysia

**Figure 5:**
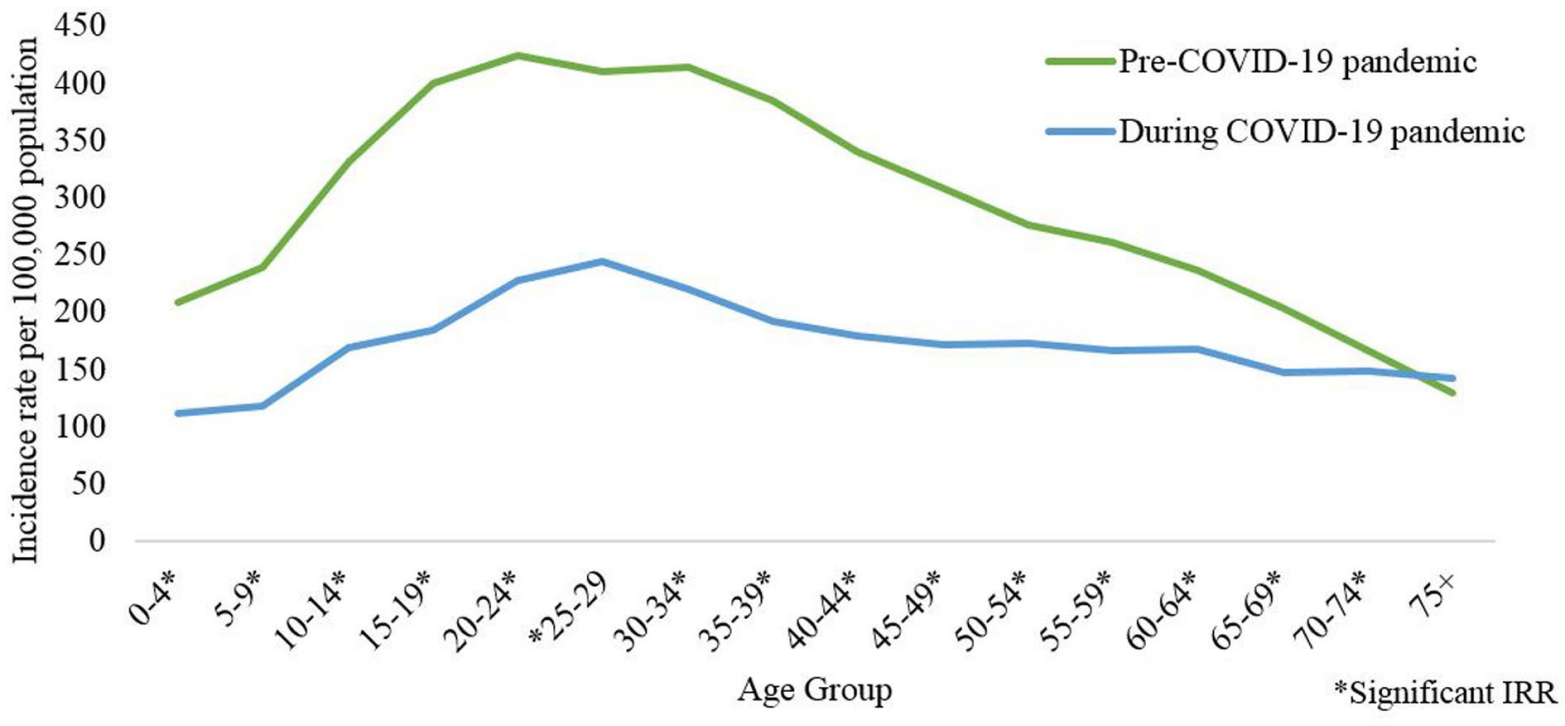
Average dengue incidence rate by age-group for the pre- (2014–2019) and during COVID-19 pandemic (2020–2021), Malaysia (per 100,000 population).

At the national level, the average age-specific dengue incidence was lowest among individuals aged 0–4 years at 209.1 and 112.3 per 100,000 population in the pre and during the COVID-19 pandemic phase, respectively. Following this as age advances there is an increase in the average dengue age-specific incidence which peaked among individuals aged 30–34 years (413.3 per 100,000 population) and aged 25–29 years (244.2 per 100,000 population) in the pre and during COVID-19 pandemic phase, respectively. Subsequently, the average dengue age-specific incidence reduced among individuals more than 34 years, reaching the lowest among individuals aged more than 75 years with 129 and 142.0 per 100,000 population in the pre and during the COVID-19 pandemic phase, respectively, as shown in Figure 5.

Overall, at the national level, a decrease in the average dengue age-specific incidence and significantly lower was observed across all age groups except 75 and above during the pandemic phase compared to the pre-pandemic phase as shown in Table 4 and Supplementary Table S1. The highest reduction in the average dengue age-specific incidence was observed within age groups 15 to 19 at 53.7%, while the lowest reduction was observed within age groups 70 to 74 at 10.6%. At the state level, only Selangor and Kelantan showed similar trends of the average dengue age-specific incidence at the national level (Table 4).

**Table 4:** Average incidence rate per 100,000 population by age-group.

State	Age group															
	0–4		5–9		10–14		15–19		20–24		25–29		30–34		35–39	
	Pre*	During**	Pre*	During**	Pre*	During**	Pre*	During**	Pre*	During**	Pre*	During**	Pre*	During**	Pre*	During**
Malaysia	**209.1**	**112.3**	**239.5**	**117.9**	**331.5**	**168.5**	**399.8**	**185.0**	**424.0**	**228.1**	**409.5**	**244.2**	**413.3**	**220.5**	**385.0**	**192.2**
Johor	**200.0**	**116.2**	**156.5**	**100.6**	**238.6**	**151.8**	**331.8**	**201.5**	**342.0**	**203.3**	**340.7**	**223.3**	**333.0**	**215.6**	**304.3**	**180.9**
Kedah	19.8	18.5	**33.6**	**15.6**	**51.4**	**28.6**	**65.3**	**36.8**	**61.4**	**34.1**	**69.8**	**33.4**	**84.4**	**45.6**	**79.6**	**44.3**
Kelantan	**120.8**	**44.3**	**201.2**	**65.3**	**364.1**	**129.5**	**429.1**	**136.8**	**364.5**	**137.1**	**405.5**	**156.9**	**430.9**	**152.3**	**380.6**	**138.4**
Melaka	100.0	109.5	**111.4**	**75.0**	177.0	146.7	**276.5**	**154.0**	**266.0**	**208.4**	**252.0**	**199.7**	**251.1**	**161.3**	**264.2**	**176.6**
Negeri Sembilan	**149.1**	**91.0**	**168.1**	**72.8**	**208.9**	**134.4**	**279.8**	**169.8**	**294.0**	**198.6**	**267.9**	**181.8**	**312.3**	**162.3**	**335.1**	**159.4**
Pahang	61.0	48.7	87.6	69.8	**129.7**	**103.1**	**161.9**	**112.8**	150.1	178.1	166.9	158.6	163.0	152.3	**167.4**	**103.2**
Perak	**178.2**	**53.7**	**252.3**	**53.4**	**384.7**	**77.5**	**470.5**	**95.9**	**326.2**	**80.1**	**292.1**	**81.8**	**312.9**	**90.3**	**304.3**	**86.3**
Perlis	4.6	0.6	**7.6**	**1.5**	**13.0**	**1.3**	**13.4**	**2.0**	**11.9**	**2.5**	**13.5**	**2.1**	**14.6**	**1.9**	**13.8**	**2.9**
Pulau Pinang	**671.5**	**143.7**	**869.7**	**100.5**	**1420.6**	**136.2**	**1728.9**	**91.1**	**1530.0**	**166.9**	**1793.5**	**270.8**	**2331.7**	**261.5**	**2739.3**	**224.8**
Sabah	47.0	42.9	**65.4**	**53.4**	**83.9**	**68.5**	**88.9**	**59.6**	**60.5**	**45.8**	46.4	42.6	36.7	33.5	**35.6**	**26.7**
Sarawak	**29.2**	**8.7**	**65.7**	**12.8**	**106.6**	**37.7**	**152.4**	**52.6**	**166.4**	**60.4**	**175.6**	**94.3**	**238.1**	**103.7**	**259.2**	**88.6**
Selangor	**1032.5**	**504.7**	**1083.4**	**565.9**	**1437.7**	**673.1**	**1360.8**	**733.8**	**1663.0**	**1012.5**	**1601.1**	**1052.1**	**1794.2**	**1124.9**	**1708.6**	**1041.4**
Terengganu	**18.6**	**4.8**	**24**	**6.9**	**38.8**	**10.4**	**55.3**	**11.8**	**53.3**	**14.3**	**51.3**	**11.3**	**51.7**	**10.2**	**41.0**	**9.0**
F.T. Kuala Lumpur	**331.9**	**226.6**	**440.6**	**283.3**	**594.2**	**324.4**	**776.1**	**390.6**	**769.1**	**631.4**	**672.3**	**489.6**	**578.5**	**382.3**	**478**	**265.8**
F.T. Labuan	35.2	10.1	54.7	-	28.7	-	62.0	18.2	57.7	-	54.1	-	49.6	17.7	29.3	5.0
F.T. Putrajaya	302.0	231.2	**391.1**	**239.4**	608.4	410.4	607.4	379.2	793.0	690.5	**1005.3**	**562.5**	**949.2**	**462.4**	**682.4**	**339.4**

State	Age group															
	40–44		45–49		50–54		55–59		60–64		65–69		70–74		75 and above	
	Pre*	During**	Pre*	During**	Pre*	During**	Pre*	During**	Pre*	During**	Pre*	During**	Pre*	During**	Pre*	During**
Malaysia	**340.4**	**178.9**	**307.5**	**171.2**	**276.1**	**173.1**	**261.4**	**166.2**	**237.2**	**168.0**	**203.0**	**147.1**	**166.3**	**148.6**	129.0	142.0
Johor	**274.6**	**170.7**	**296.4**	**159.8**	**212.4**	**157.8**	**191.0**	**137.2**	**170.4**	**134.4**	140.3	139.1	111.3	123.4	74.8	93.3
Kedah	**75.4**	**23.7**	**88.4**	**32.7**	**90.8**	**35.5**	**93.5**	**46.0**	**71.9**	**40.1**	**66.2**	**38.7**	61.0	42.2	47.4	45.3
Kelantan	**365.5**	**135.9**	**335.3**	**106.4**	**346.5**	**117.5**	**319.4**	**105.0**	**284.6**	**129.9**	**212.7**	**88.1**	**169.7**	**99.7**	90.1	52.1
Melaka	**264.7**	**122.6**	242.4	214.2	236.3	241.1	237.7	237.2	230.3	257.8	195.6	259.6	**177.7**	**295.7**	**140.1**	**269.2**
Negeri Sembilan	**303.2**	**159.7**	**322.5**	**148.9**	**235.6**	**173.2**	**219.9**	**145.5**	199.5	154.0	167.5	121.2	159.3	126.3	108.1	156.5
Pahang	**171.2**	**92.7**	**170.1**	**114.2**	**181.1**	**127.6**	162.5	130.9	148.3	164.7	125.2	125.5	98.8	100.5	73.4	71.8
Perak	**297.5**	**81.6**	**291.7**	**81.7**	**325.6**	**81.8**	**339.0**	**111.2**	**318.8**	**138.7**	**288.5**	**179.7**	249.4	215.8	226.1	264.4
Perlis	**10.6**	**1.8**	**12.5**	**3.0**	9.8	4.3	**11.0**	**2.2**	7.6	2.3	6.1	1.7	5.0	1.6	**20.6**	**0.7**
Pulau Pinang	**2746.9**	**202.7**	**2264.2**	**280.6**	**1859.3**	**414.6**	**1604.3**	**417.7**	**1395.1**	**540.7**	**1243.2**	**512.5**	**996.9**	**658.4**	**638.6**	**971.9**
Sabah	32.9	26.9	**38.3**	**29.1**	42.5	39.6	45.5	39.0	46.6	43.5	39.7	32.9	42.0	35.2	**98.8**	**52.6**
Sarawak	**265.5**	**131.0**	**261.2**	**153.6**	**238.3**	**180.8**	**231.0**	**155.8**	**240.0**	**160.1**	**218.5**	**139.8**	202.1	153.8	144.2	218.8
Selangor	**1568.1**	**1095.4**	**1425.1**	**1068.3**	**1332.2**	**1056.3**	**1327.7**	**1095.1**	**1338.6**	**1029.3**	**1338.8**	**696.2**	**1040.5**	**832.3**	672.1	638.8
Terengganu	**35.7**	**7.6**	**38.5**	**6.9**	**43.6**	**5.1**	**40.8**	**8.9**	**29.9**	**9.6**	**25.7**	**9.8**	25.6	10.8	17.3	5.1
F.T. Kuala Lumpur	**412.4**	**250.1**	**345.3**	**229.5**	300.9	272.6	298.3	280.2	268.9	290.5	236.8	238.7	175.9	231.1	**513.2**	**211.9**
F.T. Labuan	39.2	-	28.4	-	22.1	23.9	40.3	-	64.7	-	65.4	27.8	39.4	-	-	-
F.T. Putrajaya	**555.8**	**286.3**	420.4	271.1	382.7	295.5	361.5	407.9	496.5	317.0	394.7	180.6	1166.7	125.0	2055.6	125.0

*Incidence rate pre-COVID-19 pandemic. **Incidence rate during COVID-19 pandemic. Bold font indicates significant incidence rate ratio.

**Figure 6:**
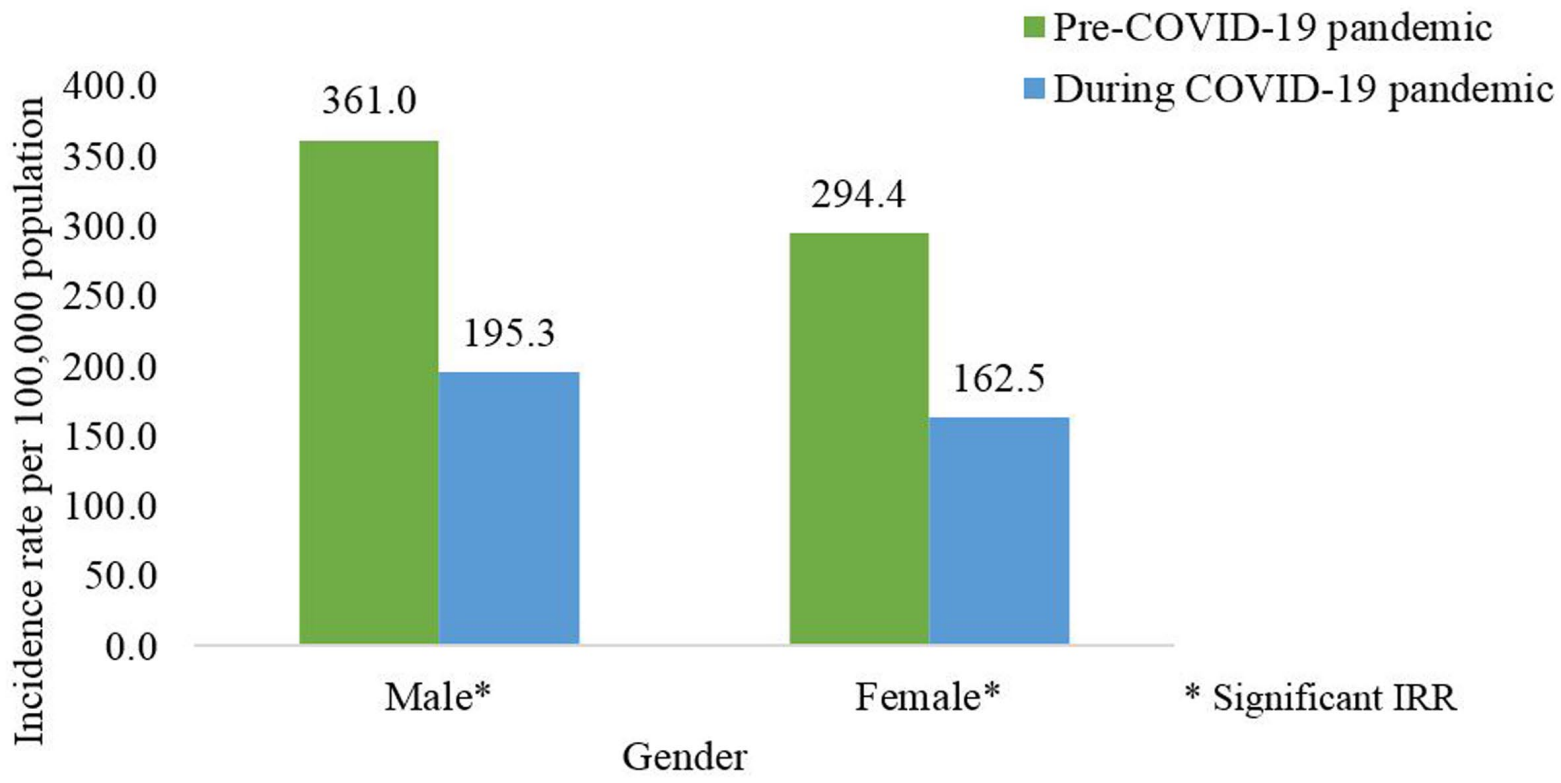
Average dengue incidence rate by gender for the pre- (2014-2019) and during COVID-19 pandemic (2020–2021), Malaysia (per 100,000 population).

At the national level, the incidence of dengue cases among males and females was significantly lower during the pandemic compared to the pre-pandemic phase as shown in Table 5 and Supplementary Table S1. Wherein the incidence of dengue cases by gender for the pre and pandemic phases was 361.0 and 195.3 per 1,000,000 males population; 294.4 and 162.5 for females, respectively, (Figure 6). At the state level, similar distributions of the incidence of dengue cases by gender were observed for all states except for Sabah which reported higher incidence among males during the pandemic phase (Table 5).

**Table 5:** Average incidence rate per 100,000 population by demographic characteristics.

State	Demographic characteristics													
	Gender				Nationality				Ethnicity					
	Male		Female		Malaysian		Non-Malaysian		Bumiputera		Chinese		Indian	
	Pre*	During**	Pre*	During**	Pre*	During**	Pre*	During**	Pre*	During**	Pre*	During**	Pre*	During**
Malaysia	**361**	**195.3**	**294.4**	**162.5**	**335.9**	**183.3**	**264.9**	**138.1**	**308.8**	**174.8**	**357.9**	**180.1**	**525.3**	**277.4**
Johor	**292.4**	**177.7**	**225.8**	**154.8**	**255.8**	**169.7**	**309.4**	**135.2**	**239.8**	**164.5**	**256.5**	**167.6**	**389.5**	**235.3**
Kedah	**69.5**	**36.5**	**58.6**	**29.9**	**65.7**	**34.5**	**28.2**	**5.6**	**56.9**	**32.2**	**86.7**	**40.3**	**126.5**	**47.7**
Kelantan	**320.6**	**114.7**	**314.5**	**114.6**	**323.6**	**116.1**	**108.4**	**45.4**	**326.2**	**115.7**	**274.8**	**128.7**	**257**	**74.1**
Melaka	**240.6**	**183.2**	**197.2**	**161.3**	**220.4**	**181.8**	**193.3**	**55**	**205.1**	**162.8**	242.9	228.2	288.5	247.1
Negeri Sembilan	**267.7**	**154.5**	**224.3**	**139.8**	**251.7**	**149**	**181.9**	**119.5**	**233.5**	**160.3**	**280**	**112.7**	**283.8**	**157.1**
Pahang	**142.2**	**115.3**	**139.3**	**113.6**	**147**	**118.6**	48.6	35.6	**138.6**	**117.3**	**179.7**	**130.2**	**192.1**	**116.3**
Perak	**229.3**	**69.1**	**202.7**	**59.5**	**217.4**	**66.6**	**185.7**	**25.9**	**168.7**	**55.2**	**266.4**	**81.1**	**331.9**	**90.6**
Perlis	**113**	**14.7**	**98.5**	**20.7**	**107.8**	**18.1**	47.9	-	**102.5**	**16.6**	**169.1**	**34.5**	155.9	19.6
Pulau Pinang	**249.3**	**44.8**	**221**	**38.3**	**241.9**	**44.1**	**162.6**	**13.7**	**190.5**	**29.6**	**287.8**	**56.7**	**270.4**	**54.4**
Sabah	92.7	95.3	76	74.8	**114.1**	**104.9**	**1746.4**	**22**	**102.9**	**87.9**	142.1	164.3	146.4	260.6
Sarawak	**77.2**	**48**	**64.7**	**30.5**	**72.8**	**39.7**	47.5	37.3	**57.1**	**41.8**	**122.6**	**31.7**	48.4	88.5
Selangor	**980.1**	**468.1**	**766.8**	**389.8**	**881.6**	**436.7**	**843.4**	**366.1**	**945.6**	**475**	**728.1**	**332**	**906.1**	**499.1**
Terengganu	**98.7**	**21.2**	**86.3**	**18**	**93.5**	**20.1**	**62.3**	**1.9**	**91.6**	**20**	**171.3**	**30.2**	90	-
F.T. Kuala Lumpur	**525.7**	**351.2**	**453.1**	**299**	**507.6**	**321.5**	375.1	373.4	**608.6**	**397.2**	**408**	**249**	**472.8**	**268.1**
F.T. Labuan	**47.8**	**7.1**	**38.7**	**5.4**	**46.9**	**6.5**	14.1	4.7	**44.7**	**6.9**	33.2	5.1	65.5	-
F.T. Putrajaya	**624.6**	**341.1**	**534.7**	**356.4**	**588.2**	**353.7**	256.8	174.2	**590.7**	**357.6**	584.1	-	597.7	269.3

*Pre-COVID-19 pandemic. **During COVID-19 pandemic. Bold font indicates significant incidence rate ratio.

**Figure 7:**
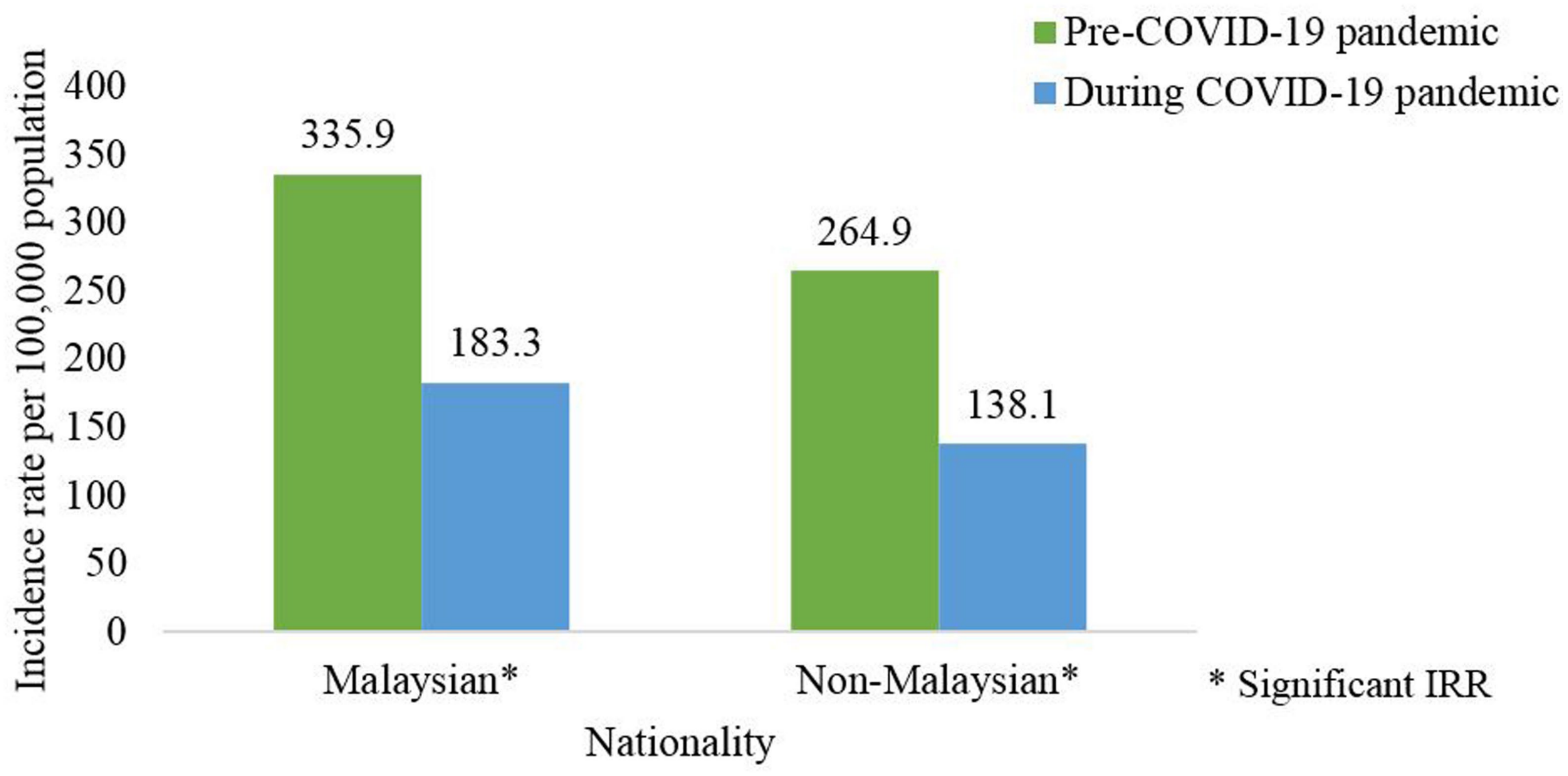
Average dengue incidence rate among nationality for the pre- (2014–2019) and during COVID-19 pandemic (2020–2021), Malaysia (per 100,000 population).

At the national level, the incidence of dengue cases among Malaysian and non-Malaysians was significantly lower during the pandemic compared to the pre-pandemic phase as shown in Table 5 and Supplementary Table S1. Wherein the incidence of dengue cases by nationality for the pre and pandemic phases was 335.9 and 183.3 per 100,000 for the Malaysian population; 264.9 and 138.1 for non-Malaysians, respectively, (Figure 7). At the state level, similar distributions of the dengue incidence among Malaysian were observed for all states during both phases (Table 5).

**Figure 8:**
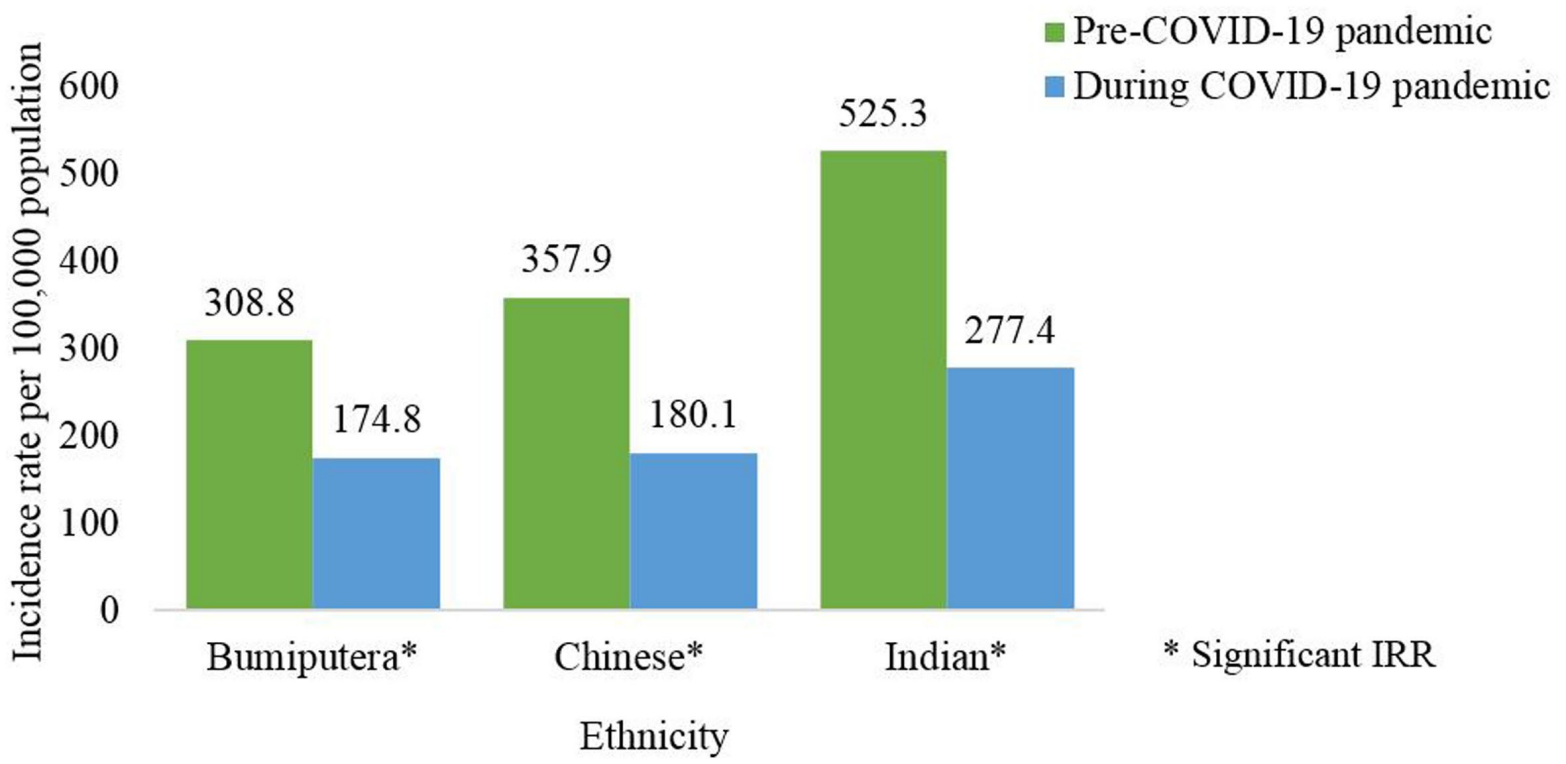
Average dengue incidence rate among ethnicity for the pre- (2014–2019) and during COVID-19 pandemic (2020–2021), Malaysia (per 100,000 population).

At the national level, the incidence of dengue cases among Bumiputera, Chinese and Indians were significantly lower during the pandemic compared to the pre-pandemic phase (Supplementary Table S1). The average incidence rate of dengue cases among Indians was higher compared to the other ethnic groups during the pre and pandemic phases. Wherein the incidence rate of dengue cases per 100,000 population by ethnicity for the pre and pandemic phases was 308.8 and 174.8 for Bumiputera; 357.9 and 180.1 for Chinese and 525.3 and 277.4 for Indians (Figure 8). At the state level, similar distributions of the dengue incidence among Bumiputera were observed for all states during both phases (Table 5).

## 4. Discussion

During the COVID-19 pandemic, there was an associated reduction of infectious diseases such as dengue, malaria, hand, foot and mouth disease, hepatitis E, malaria, influenza, and scarlet fever (3, 5, 18). This reduction can be attributed to the implementation of Public Health and Social Measures (PHSM) strategies to control the COVID-19 pandemic which in turn reduced transmission of other infectious diseases (19–21). In addition, disease under-reporting may have contributed to lower reporting of other infectious diseases during the pandemic due to resource constraints and a decrease in access to healthcare services (22). Furthermore, as dengue and COVID-19 infections have similar common clinical presentation (i.e., fever, headache, cough and myalgia) and immune responses these would result in the misdiagnosis of these two infections (23, 24). Also, studies have reported potential co-infection and cross-reaction between dengue and COVID-19 infections which resulted in false-positive results (25–28).

Dengue incidence in Malaysia peaked in the years 2015 and 2019. Similar dengue trends have been observed in the year 2019 among neighboring countries such as Cambodia, Indonesia, Philippines, Thailand, and Vietnam (29). Following the peak in 2015, dengue incidence gradually reduced by 17 to 36% in the subsequent years from 2016 to 2018. However, after the 2019 peak, the dengue incidence had markedly reduced by 30 and 80% in the years 2020 and 2021, respectively, compared to 2019. This drastic reduction was beyond the reduction usually observed post-peak and is attributed to the COVID-19 pandemic which coincided in the years following 2019. This is supported by the strong negative correlation of 0.901 between the weekly number of dengue and COVID-19 cases during the pandemic phase as reported in this study. However, several states showed decrease in dengue cases in the year 2015 and 2019, which could be attributed to differences in vector distribution, viral serotypes, population density and environmental factors. Therefore, resulting in variations of dengue cases in different regions (30, 31).

In this study, we found there was a reduction of average dengue cases by 44.0% during the COVID-19 pandemic compared to the pre-pandemic phase. A similar finding was reported in several Southeast Asian and Latin American countries which found the reduction of dengue infection ranging from 30 to 70% during the COVID-19 pandemic (32). The reduction of dengue infection during the pandemic was a result of the measures imposed to control the pandemic which included the institution of PHSM. More specifically measures such as physical distancing, movement restriction and closure of most institutes/workplaces would influence the transmission of vector-borne diseases like dengue by reducing the risk of transmission. Wherein movement restrictions would limit population mobility, therefore, reducing the risk of exposure to infected vectors. In addition, movement restrictions and fear of contacting COVID-19 may hinder individuals from getting tested in health facilities, therefore, resulting in under-reporting (33, 34).

This study found that the average dengue incidence rate had reduced in the majority of the states (15 out of 16 states) and districts (101 out of 145 districts) during the COVID-19 pandemic compared to the pre-pandemic phase. Wherein the highest reduction in dengue incidence was reported in Selangor state (446.4) and Petaling district (548.0). Among the reasons that could have attributed to this finding was the high COVID-19 burden in areas that were highly populous, dense and urbanized. Several studies in Malaysia showed an inverse relationship between annual cases and incidence of dengue and COVID-19 for the year 2020 and following the implementation of the movement control order (9, 10). Furthermore, areas that had a high COVID-19 burden had stricter, extensive and prolonged PHSM which in turn resulted in lower dengue incidence (35, 36).

In addition, this study compared the average dengue incidence during the COVID-19 pandemic and pre-pandemic phases in different demographic subgroups. During the COVID-19 pandemic, the age-specific dengue incidence rate was consistently lower in all age groups compared to the pre-pandemic phase. Wherein, the highest reduction was observed among individuals aged 20 to 35 years. This finding could be attributed to the reduction in outdoor activities among individuals aged between 20 to 35 years as a result of the PHSM during the COVID-19 pandemic (37). Furthermore, the learning institute remained closed during the movement restriction order and dengue among school-going children (age group between 5 to 14 years) was notably lower. This portion may be due to peridomestic transmission whereas the reduced portion may be accounted for transmission that takes place en route to school / within the school. Thus, the control activity needs to continue in school to disrupt dengue transmission.

The average dengue incidence among males was higher than females during the pre and pandemic phases. During the pandemic phase, more males were infected with dengue and this could be attributed to the larger proportion of males being involved as COVID-19 frontliners, i.e., police force, army and food deliveries, therefore increasing the risk of dengue transmission compared to females who limited their mobility due to caring for children when schools were closed during COVID-19 pandemic (37). Dengue incidence was consistently higher for the pre-pandemic compared to the pandemic phase across both genders except for males in Sabah. This finding could be attributed due to population behavioral factors which could result in non-compliance to movement control measures and inadequate monitoring of population mobility in this state as reported in a previous study (38). The average dengue incidence among Malaysians was higher than non-Malaysians during the pre and pandemic phases, however, there were no notable differences in the nationality across both phases. In addition, all ethnic groups showed a reduction in dengue incidence pre and during the pandemic with the highest reduction being observed among the Indian ethnic group. Certain ethnic groups are commonly involved in outdoor laborious work and therefore during the pandemic the PHSM limited their outdoor activities and resulting in lower dengue incidence (20, 34).

There are several strengths to this study. First, to date, there are no studies on the distribution of dengue cases during the pre and COVID-19 pandemic phases in Malaysia. Therefore, to address this gap in the literature, the pandemic period included in this study was the years 2020 and 2021 which comprehensively covered the entire pandemic duration. In addition, this study describes the effect of the COVID-19 pandemic on dengue cases by evaluating the correlation between weekly dengue cases and COVID-19 cases at the national and state levels. More specifically this study provides valuable information to health authorities in identifying geographical areas (state and district) which reported lower/higher dengue cases during the pandemic phase as well as variation in dengue demographics-related factors (age, gender, nationality and ethnicity). These study objectives were limited to the geographical (state and district) and demographic characteristics (age, gender, nationality and ethnicity), however other factors such as virological (i.e., viral interaction, dengue serotype shift) and environmental (i.e., temperature, humidity, rainfall) were not accounted in this study. Future studies should take to account these factors as they could have affected the transmission of dengue trends in Malaysia.

## 5. Conclusion

This study provides evidence that the COVID-19 pandemic had affected dengue case trends in Malaysia. Wherein there was a drastic decline in dengue incidence during the COVID-19 pandemic from 2020 to 2021. Furthermore, the distribution of dengue cases and incidence by demographic sub-groups (gender, nationality, ethnicity) were higher during the pre-COVID-19 pandemic compared to the pandemic phase. Currently, as COVID-19 transition into the endemic phase, we would expect a resurgence of dengue cases especially in areas which reported lower cases during the pandemic phase. Overall, this study assisted in generating hypotheses for further in-depth studies to determine the specific factors driving the reduction in dengue during the pandemic phase.

## Data availability statement

The datasets presented in this article are not readily available because restrictions apply to the availability of these data for this study. Data were obtained with the permission of the Ministry of Health Malaysia. Requests to access the datasets should be directed to Ministry of Health Malaysia.

## Author contributions

NHM, BG, and SS conceived and planned the study. AZ, AM, CT, NN, LA, and MK retrieved and managed the data. NHM, SG, CL, NG, ML, and MM analyzed the data. SS, JJ, NM, MW, and BG critically reviewed the draft manuscript. All authors discussed the results and contributed to the final manuscript.

## Funding

This study was funded by the authors’ own institution which supported the conduct of the study and publication fee. The funders had no role in the study design, analysis and preparation of the manuscript.

## Conflict of interest

The authors declare that the research was conducted in the absence of any commercial or financial relationships that could be construed as a potential conflict of interest.

## Publisher’s note

All claims expressed in this article are solely those of the authors and do not necessarily represent those of their affiliated organizations, or those of the publisher, the editors and the reviewers. Any product that may be evaluated in this article, or claim that may be made by its manufacturer, is not guaranteed or endorsed by the publisher.
