# Supplementary material for: The effects of the COVID-19 pandemic on dengue cases in Malaysia
Source: Front Public Health. 2023 Aug 24;11:1213514. doi: 10.3389/fpubh.2023.1213514 (PMC10484591; doi:10.3389/fpubh.2023.1213514)
Supplement: Supplementary file 1 [file Table_1.PDF]

Table 1 Incidence rate ratio by demographic characteristics

	Age group<br>Incidence Rate Ratio (95% Confidence Interval)															
State	0-4	5-9	10-14	15-19	20-24	25-29	30-34	35-39	40-44	45-49	50-54	55-59	60-64	65-69	70-74	>75
Malaysia	0.54 (0.51,0.56)	0.49 (0.47,0.51)	0.51 (0.49,0.53)	0.46 (0.45,0.48)	0.54 (0.52,0.55)	0.6 (0.58,0.61)	0.53 (0.52,0.55)	0.5 (0.48,0.52)	0.53 (0.51,0.55)	0.56 (0.53,0.58)	0.63 (0.6,0.66)	0.64 (0.6,0.67)	0.71 (0.67,0.75)	0.72 (0.67,0.78)	0.89 (0.81,0.98)	1.1 (1.00,1.21)
Johor	0.58 (0.51,0.66)	0.64 (0.56,0.74)	0.64 (0.57,0.71)	0.61 (0.55,0.67)	0.59 (0.54,0.65)	0.66 (0.6,0.72)	0.65 (0.59,0.71)	0.59 (0.53,0.66)	0.62 (0.55,0.7)	0.54 (0.47,0.61)	0.74 (0.64,0.86)	0.72 (0.61,0.85)	0.79 (0.65,0.95)	0.99 (0.78,1.26)	1.11 (0.81,1.53)	1.25 (0.89,1.74)
Kedah	0.93 (0.58,1.5)	0.46 (0.3,0.73)	0.56 (0.39,0.79)	0.56 (0.42,0.75)	0.56 (0.42,0.74)	0.48 (0.36,0.64)	0.54 (0.4,0.72)	0.56 (0.4,0.77)	0.31 (0.21,0.48)	0.37 (0.25,0.54)	0.39 (0.27,0.57)	0.49 (0.35,0.7)	0.56 (0.37,0.84)	0.58 (0.36,0.95)	0.69 (0.38,1.24)	0.96 (0.55,1.65)
Kelantan	0.37 (0.28,0.47)	0.32 (0.26,0.4)	0.36 (0.31,0.41)	0.32 (0.28,0.36)	0.38 (0.32,0.44)	0.39 (0.33,0.45)	0.35 (0.3,0.42)	0.36 (0.3,0.44)	0.37 (0.3,0.46)	0.32 (0.25,0.4)	0.34 (0.27,0.43)	0.33 (0.26,0.42)	0.46 (0.35,0.59)	0.41 (0.29,0.6)	0.59 (0.38,0.91)	0.58 (0.33,1.03)
Melaka	1.1 (0.8,1.5)	0.67 (0.48,0.94)	0.83 (0.64,1.08)	0.56 (0.45,0.68)	0.78 (0.65,0.94)	0.79 (0.65,0.97)	0.64 (0.52,0.79)	0.67 (0.53,0.85)	0.46 (0.36,0.6)	0.88 (0.68,1.15)	1.02 (0.78,1.34)	1 (0.76,1.32)	1.12 (0.83,1.52)	1.33 (0.93,1.89)	1.66 (1.07,2.59)	1.92 (1.24,2.99)
Negeri Sembilan	0.61 (0.46,0.8)	0.43 (0.33,0.57)	0.64 (0.51,0.8)	0.61 (0.5,0.73)	0.68 (0.57,0.8)	0.68 (0.56,0.82)	0.52 (0.43,0.63)	0.48 (0.39,0.58)	0.53 (0.42,0.66)	0.46 (0.36,0.59)	0.74 (0.56,0.96)	0.66 (0.5,0.87)	0.77 (0.57,1.05)	0.72 (0.49,1.07)	0.79 (0.48,1.31)	1.45 (0.88,2.38)
Pahang	0.8 (0.58,1.1)	0.8 (0.61,1.04)	0.79 (0.64,0.98)	0.7 (0.57,0.84)	1.19 (0.99,1.42)	0.95 (0.79,1.15)	0.93 (0.77,1.13)	0.62 (0.49,0.77)	0.54 (0.42,0.69)	0.67 (0.52,0.87)	0.7 (0.54,0.92)	0.81 (0.61,1.06)	1.11 (0.82,1.51)	1 (0.68,1.47)	1.02 (0.61,1.71)	0.98 (0.56,1.71)
Perak	0.3 (0.23,0.4)	0.21 (0.16,0.28)	0.2 (0.16,0.25)	0.2 (0.17,0.25)	0.25 (0.2,0.3)	0.28 (0.23,0.34)	0.29 (0.24,0.35)	0.28 (0.23,0.35)	0.27 (0.22,0.34)	0.28 (0.22,0.35)	0.25 (0.2,0.32)	0.33 (0.26,0.41)	0.44 (0.35,0.55)	0.62 (0.48,0.8)	0.87 (0.63,1.18)	1.17 (0.87,1.57)
Perlis	0.13 (0.02,1.04)	0.2 (0.06,0.69)	0.1 (0.03,0.37)	0.15 (0.06,0.4)	0.21 (0.08,0.52)	0.16 (0.05,0.44)	0.13 (0.04,0.4)	0.21 (0.08,0.54)	0.17 (0.05,0.59)	0.24 (0.08,0.71)	0.44 (0.16,1.18)	0.2 (0.06,0.69)	0.3 (0.07,1.24)	0.28 (0.05,1.69)	0.32 (0.03,3.03)	0.03 (0,0.64)
Pulau Pinang	0.21 (0.14,0.32)	0.12 (0.07,0.18)	0.1 (0.06,0.14)	0.05 (0.04,0.08)	0.11 (0.08,0.14)	0.15 (0.11,0.2)	0.11 (0.08,0.15)	0.08 (0.06,0.11)	0.07 (0.06,0.1)	0.12 (0.09,0.17)	0.22 (0.17,0.3)	0.26 (0.19,0.35)	0.39 (0.29,0.53)	0.41 (0.29,0.58)	0.66 (0.44,0.99)	1.52 (1.08,2.14)
Sabah	0.91 (0.76,1.09)	0.82 (0.7,0.96)	0.82 (0.71,0.95)	0.67 (0.58,0.78)	0.76 (0.64,0.89)	0.92 (0.78,1.08)	0.91 (0.77,1.09)	0.75 (0.61,0.92)	0.82 (0.65,1.03)	0.76 (0.6,0.97)	0.93 (0.74,1.18)	0.86 (0.66,1.11)	0.93 (0.7,1.24)	0.83 (0.58,1.18)	0.84 (0.54,1.3)	0.53 (0.36,0.78)
Sarawak	0.3 (0.15,0.61)	0.19 (0.11,0.34)	0.35 (0.25,0.5)	0.35 (0.26,0.46)	0.36 (0.28,0.48)	0.54 (0.42,0.69)	0.44 (0.34,0.56)	0.34 (0.26,0.45)	0.49 (0.38,0.64)	0.59 (0.45,0.76)	0.76 (0.58,0.99)	0.67 (0.5,0.9)	0.67 (0.48,0.92)	0.64 (0.43,0.96)	0.76 (0.46,1.26)	1.52 (0.96,2.4)
Selangor	0.49 (0.46,0.52)	0.52 (0.49,0.55)	0.47 (0.44,0.49)	0.54 (0.51,0.56)	0.61 (0.59,0.63)	0.66 (0.63,0.68)	0.63 (0.6,0.65)	0.61 (0.58,0.64)	0.7 (0.66,0.74)	0.75 (0.7,0.8)	0.79 (0.74,0.85)	0.82 (0.76,0.89)	0.77 (0.7,0.84)	0.52 (0.47,0.58)	0.8 (0.69,0.93)	0.95 (0.82,1.1)
Terengganu	0.26 (0.12,0.56)	0.29 (0.16,0.53)	0.27 (0.17,0.43)	0.21 (0.14,0.33)	0.27 (0.18,0.4)	0.22 (0.14,0.34)	0.2 (0.12,0.31)	0.22 (0.13,0.37)	0.21 (0.12,0.39)	0.18 (0.09,0.35)	0.12 (0.05,0.26)	0.22 (0.11,0.42)	0.32 (0.15,0.67)	0.38 (0.16,0.9)	0.42 (0.15,1.15)	0.29 (0.08,1.09)
F.T. Kuala Lumpur	0.68 (0.59,0.79)	0.64 (0.56,0.74)	0.55 (0.49,0.61)	0.5 (0.45,0.56)	0.82 (0.75,0.9)	0.73 (0.67,0.79)	0.66 (0.6,0.72)	0.56 (0.5,0.62)	0.61 (0.54,0.69)	0.66 (0.57,0.77)	0.91 (0.76,1.07)	0.94 (0.78,1.13)	1.08 (0.87,1.34)	1.01 (0.78,1.3)	1.31 (0.94,1.84)	0.41 (0.3,0.57)
F.T. Labuan	0.29 (0.03,2.72)	-	-	0.29 (0.05,1.82)	-	-	0.36 (0.06,2.2)	0.17 (0.01,3.5)	-	-	1.08 (0.07,17.29)	-	-	0.43 (0.01,12.67)	-	-
F.T. Putrajaya	0.77 (0.48,1.22)	0.61 (0.4,0.93)	0.67 (0.45,1.01)	0.62 (0.37,1.06)	0.87 (0.54,1.4)	0.56 (0.37,0.86)	0.49 (0.35,0.68)	0.5 (0.36,0.7)	0.52 (0.31,0.86)	0.64 (0.3,1.37)	0.77 (0.33,1.81)	1.13 (0.47,2.69)	0.64 (0.2,2.01)	0.46 (0.06,3.25)	0.11 (0,2.38)	0.06 (0,1.39)

Table 1 Incidence rate ratio by demographic characteristics (continue)

	Incidence Rate Ratio (95% Confidence Interval)						
	Gender		Nationality		Ethnicity		
	**Male**	**Female**	**Malaysian**	**Non-Malaysian**	**Bumiputera**	**Chinese**	**Indian**
Malaysia	0.54 (0.53,0.55)	0.55 (0.54,0.56)	0.55 (0.54,0.56)	0.52 (0.5,0.54)	0.57 (0.56,0.58)	0.5 (0.49,0.51)	0.53 (0.51,0.55)
Johor	0.61 (0.59,0.64)	0.69 (0.66,0.72)	0.66 (0.64,0.68)	0.44 (0.39,0.49)	0.69 (0.66,0.72)	0.65 (0.61,0.69)	0.6 (0.54,0.67)
Kedah	0.53 (0.47,0.6)	0.51 (0.45,0.58)	0.53 (0.48,0.58)	0.2 (0.08,0.52)	0.57 (0.51,0.63)	0.46 (0.36,0.58)	0.38 (0.28,0.51)
Kelantan	0.36 (0.34,0.39)	0.36 (0.34,0.39)	0.36 (0.34,0.38)	0.42 (0.24,0.73)	0.35 (0.33,0.37)	0.47 (0.35,0.64)	0.29 (0.09,0.9)
Melaka	0.76 (0.7,0.83)	0.82 (0.74,0.9)	0.82 (0.77,0.88)	0.28 (0.19,0.41)	0.79 (0.73,0.86)	0.94 (0.83,1.06)	0.86 (0.68,1.09)
Negeri Sembilan	0.58 (0.54,0.63)	0.62 (0.57,0.68)	0.59 (0.55,0.63)	0.66 (0.5,0.87)	0.69 (0.64,0.75)	0.4 (0.35,0.46)	0.55 (0.47,0.64)
Pahang	0.81 (0.74,0.88)	0.82 (0.75,0.9)	0.81 (0.76,0.86)	0.73 (0.46,1.16)	0.85 (0.79,0.91)	0.72 (0.62,0.84)	0.61 (0.45,0.82)
Perak	0.3 (0.28,0.32)	0.29 (0.27,0.32)	0.31 (0.29,0.33)	0.14 (0.1,0.2)	0.33 (0.3,0.36)	0.3 (0.27,0.33)	0.27 (0.23,0.31)
Perlis	0.13 (0.08,0.21)	0.21 (0.14,0.31)	0.17 (0.13,0.23)	-	0.16 (0.11,0.22)	0.2 (0.09,0.45)	0.13 (0.02,1.11)
Pulau Pinang	0.18 (0.16,0.2)	0.17 (0.15,0.19)	0.18 (0.17,0.2)	0.08 (0.05,0.13)	0.16 (0.14,0.18)	0.2 (0.18,0.22)	0.2 (0.16,0.25)
Sabah	1.03 (0.96,1.1)	0.98 (0.91,1.06)	0.92 (0.87,0.97)	0.01 (0.01,0.01)	0.85 (0.8,0.9)	1.16 (1.01,1.33)	1.78 (0.92,3.46)
Sarawak	0.62 (0.56,0.68)	0.47 (0.42,0.53)	0.55 (0.51,0.6)	0.79 (0.55,1.13)	0.73 (0.67,0.8)	0.26 (0.22,0.31)	1.83 (0.49,6.81)
Selangor	0.48 (0.47,0.49)	0.51 (0.5,0.52)	0.5 (0.49,0.51)	0.43 (0.41,0.45)	0.5 (0.49,0.51)	0.46 (0.45,0.47)	0.55 (0.53,0.57)
Terengganu	0.21 (0.17,0.25)	0.21 (0.17,0.26)	0.21 (0.18,0.24)	0.03 (0,0.22)	0.22 (0.19,0.25)	0.18 (0.08,0.4)	-
F.T. Kuala Lumpur	0.67 (0.64,0.7)	0.66 (0.63,0.69)	0.63 (0.61,0.65)	1 (0.91,1.1)	0.65 (0.62,0.68)	0.61 (0.57,0.65)	0.57 (0.51,0.64)
F.T. Labuan	0.15 (0.05,0.43)	0.14 (0.04,0.47)	0.14 (0.06,0.33)	0.33 (0.03,3.64)	0.15 (0.06,0.38)	0.15 (0.02,1.44)	-
F.T. Putrajaya	0.55 (0.46,0.66)	0.67 (0.56,0.81)	0.6 (0.53,0.68)	0.68 (0.22,2.08)	0.61 (0.53,0.7)	-	0.45 (0.14,1.49)
